# Supplementary material for: dsRNA formation leads to preferential nuclear export and gene expression
Source: Nature. 2024 Jun 19;631(8020):432–8. doi: 10.1038/s41586-024-07576-w (PMC11236707; doi:10.1038/s41586-024-07576-w)
Supplement: Supplementary file 1 — Supplementary Information [file 41586_2024_7576_MOESM1_ESM.pdf]

nature portfolio

https://doi.org/10.1038/s41586-024-07576-w

Supplementary information

dsRNA formation leads to preferential nuclear export and gene expression

In the format provided by the authors and unedited

SI Guide

dsRNA formation leads to preferential nuclear export and gene expression

Ivo Coban[1], Jan-Philipp Lamping[1], Anna Greta Hirsch[1], Sarah Wasilewski[1], Orr Shomroni[2], Oliver Giesbrecht[1], Gabriela Salinas[2] and Heike Krebber[1*]

[1]Abteilung für Molekulare Genetik, Institut für Mikrobiologie und Genetik, Göttinger Zentrum für Molekulare Biowissenschaften (GZMB), Georg-August Universität Göttingen, Göttingen, Germany

[2]NGS- Integrative Genomics Core Unit, University Medical Center Göttingen, Georg-August Universität Göttingen, Göttingen, Germany

[*]Correspondence: heike.krebber@biologie.uni-goettingen.de

Table of content:

SI Figure 1 Uncropped Western blots from Main Figures. (a) Western blot of Extended Figure 1a from cytoplasmic fractionation experiment for RNA-sequencing. The cytoplasmic protein Zwf1 is present in the total cell lysate and in the cytoplasmic fraction. The nuclear Yra1 and the nucleolar Nop1 are only present in the total cell lysate and absent in the cytoplasmic fraction. (**b)** Same blot as in (a) but with longer exposure time. **(c)** Western blot of Extended Figure 4b from cytoplasmic fractionation experiment in wild type cells with and without *asPHO85* expression. The cytoplasmic protein Zwf1 is present in the total cell lysate and in the cytoplasmic fraction. The nucleolar Nop1 is only present in the total cell lysate and absent in the cytoplasmic fraction. (**d)** Same blot as in (c) but with longer exposure time.

SI Figure 2 Uncropped Western blots and J2 dot-blot from Main Figures. (a) J2 dot-blot of Figure 2d showing dsRNA amounts of Wild type, Wild type treated with cycloheximide, *nmd3-2* and *rpl10(G161D)* cells. **(b)** Western blot of Figure 2f showing Protein levels of Pho85-GFP and Hem15 as negative control in *PHO85-GFP* strain after *asPHO85* induction at indicated time points. **(c)** Same Western blot as in (b) but with lower exposure time and overlayed ladder. **(d)** Western blot of Extended Figure 6e showing Rps2-GFP pulldown of RIP experiments in *mex67-5* cells. Aco1 serves as negative control. **(e)** Image of membrane (c) with external light to capture the ladder.

SI Figure 3 Uncropped EMSAs from Main Figures. (a) EMSA of Figure 2g showing shift of ssRNA after addition of recombinant Mex67-Mtr2. **(b)** EMSA of Figure 2h showing shift of dsRNA after addition of recombinant Mex67-Mtr2. **(c)** EMSA of Figure 2i showing competing Mex67-Mtr2 prebound CY5 labeled ssRNA to FAM labeled dsRNA. **(d)** EMSA of Figure 2j showing competing Mex67-Mtr2 prebound FAM labeled dsRNA to CY5 labeled ssRNA.

SI Figure 4 Uncropped EMSAs from Main Figures. (a) EMSA of Extended Figure 7f showing FAM labeled and HD-Green stained RNAs. **(b)** EMSA of Extended Figure 6f showing CY5 labeled RNAs. **(a)** Merge image of (a) and (b). **(d)** EMSA of Figure Extended 6g showing competing Mex67-Mtr2 prebound FAM labeled ssRNA to CY5 labeled dsRNA. **(e)** EMSA of Extended Figure 7h showing competing Mex67-Mtr2 prebound CY5 labeled dsRNA to FAM labeled ssRNA.

SI Figure 5 Uncropped Western blots and J2 dot-blot from Main Figures. (a) J2 dot-blot of Extended Figure 8c showing dsRNA amounts of Wild type *set2*Δ cells. **(b)** Western blot of Extended Figure 8f from cytoplasmic fractionation experiment of Wild type and *set2*Δ cells. The cytoplasmic protein Zwf1 is present in the total cell lysate and in the cytoplasmic fraction. The nucleolar Nop1 is only present in the total cell lysate and absent in the cytoplasmic fraction. (**c**) J2 dot-blot of Extended Figure 8h showing dsRNA amounts of Wild type cells with and without RNaseIII treatment. **(d)** J2 dot-blot of Extended Figure 8n showing dsRNA amounts of rpl10(G161D) cells grown at 25°C and 37°C with and without expression of *RNAseIII-NES*.

SI Figure 6 Uncropped Western blots and J2 dot-blot from Main Figures. (a) J2 dot-blot of Figure 4d showing dsRNA amounts of Wild type, *dbp2*Δ and *mtr4(G667D)* cells. **(b)** Western blot of Figure 4e showing Dbp2 coprecipitation after J2-IP with an without RNaseIII treatment. Grx4 serves as a negative control. **(c)** Western blot of Extended Figure 9f from cytoplasmic fractionation experiment of Wild type and *dbp2*Δ cells either expressing asPHO85 or not. The cytoplasmic protein Zwf1 is present in the total cell lysate and in the cytoplasmic fraction. The nucleolar Nop1 is only present in the total cell lysate and absent in the cytoplasmic fraction. **(d)** Image of membrane (c) with external light to capture the ladder.

SI Table 1: Yeast strain

SI Table 2: Plasmids

SI Table 3: qPCR primer

SI Table 4: RNA-oligos used in EMSA

SI Table 5: probes used in FISH

a

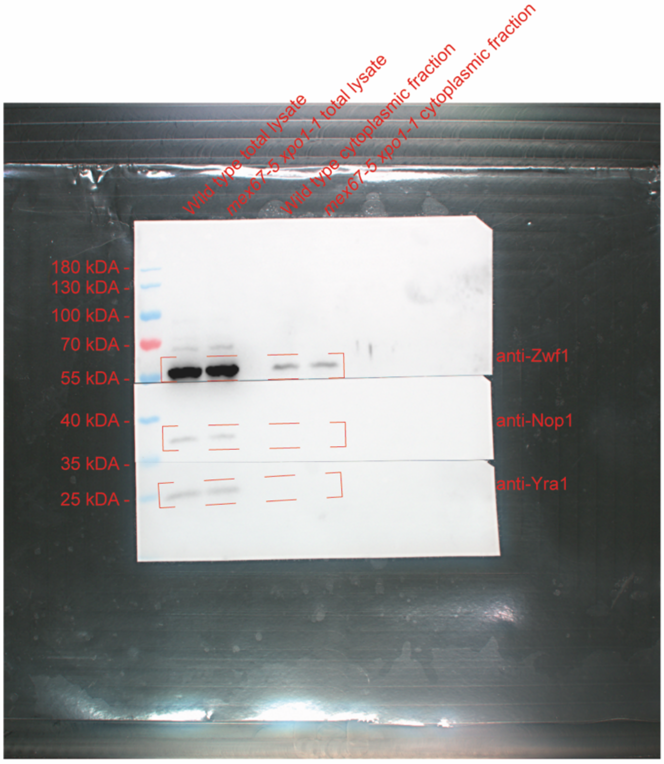

b

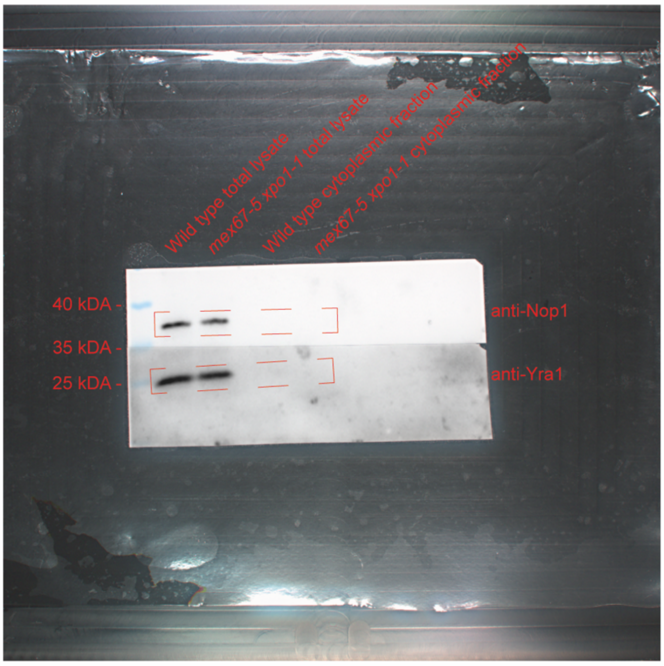

Used in Extended Figure 1a

c

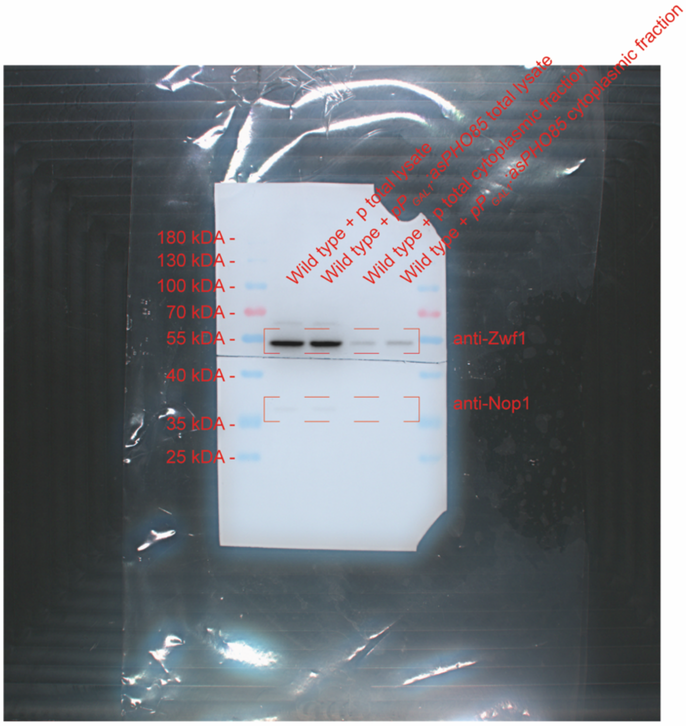

d

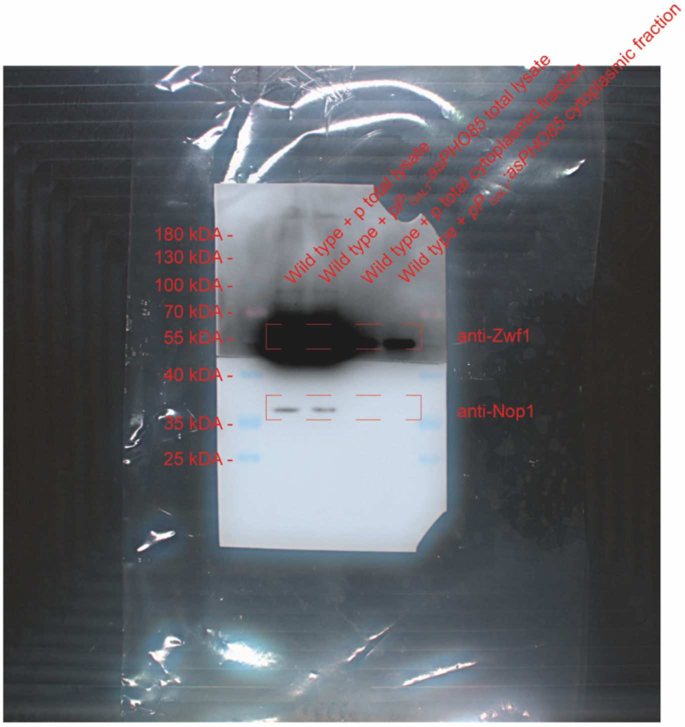

Used in Extended Figure 4b

Coban et al., SI Figure 2

a

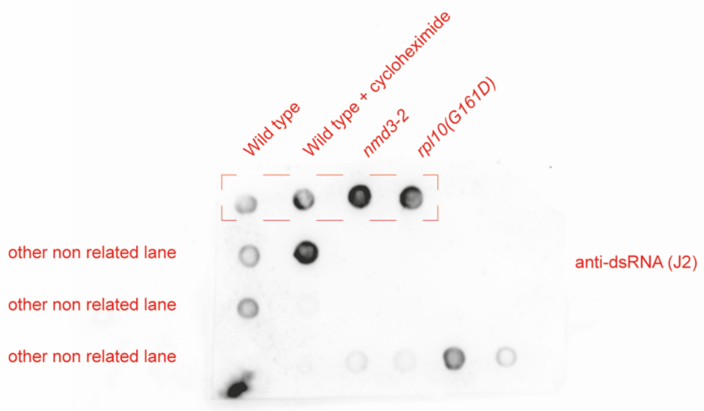

Used in Figure 2d

b

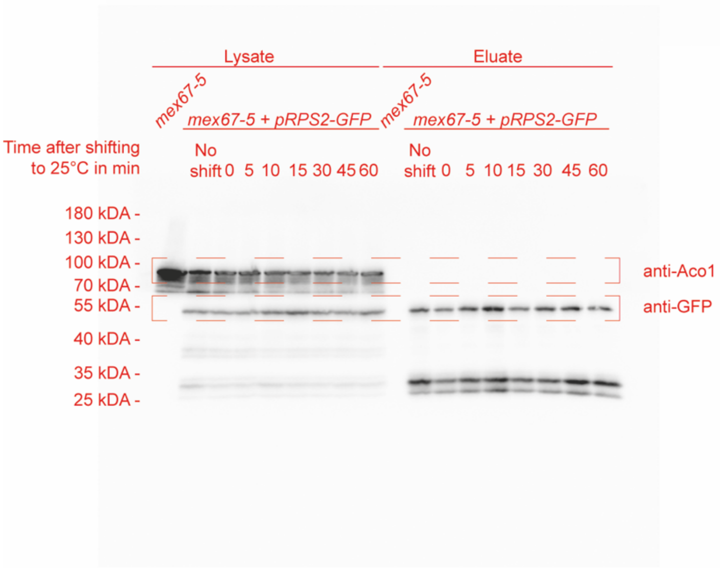

c

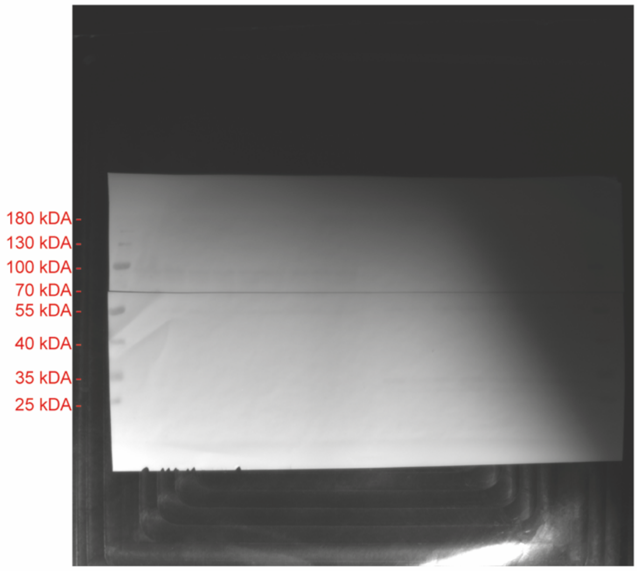

Used in Extended Figure 7e

d

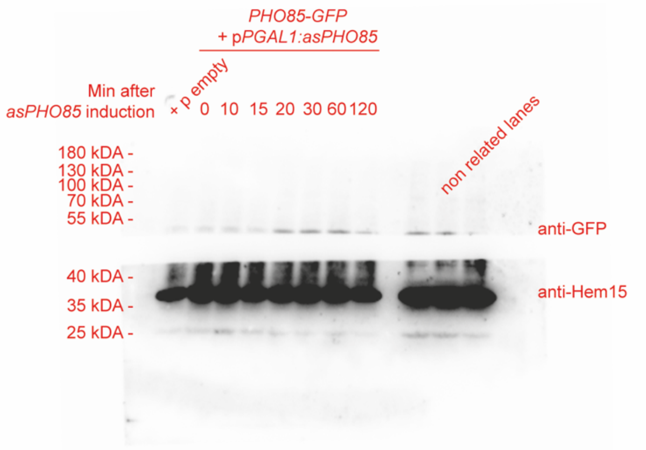

e

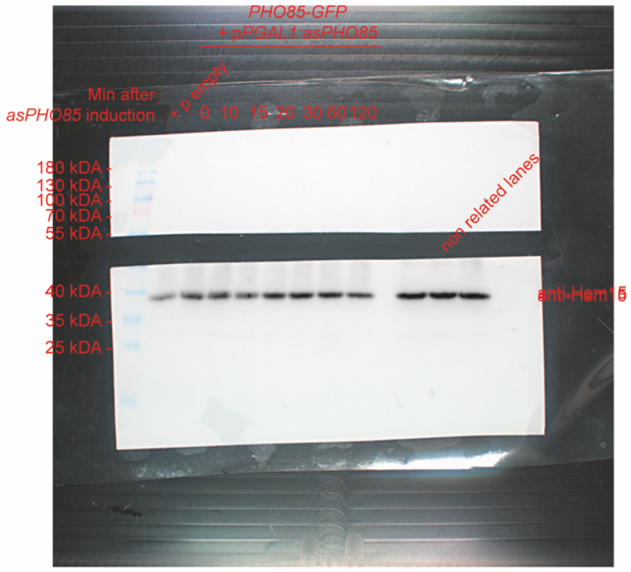

Used in Figure 2f

Coban et al., SI Figure 3

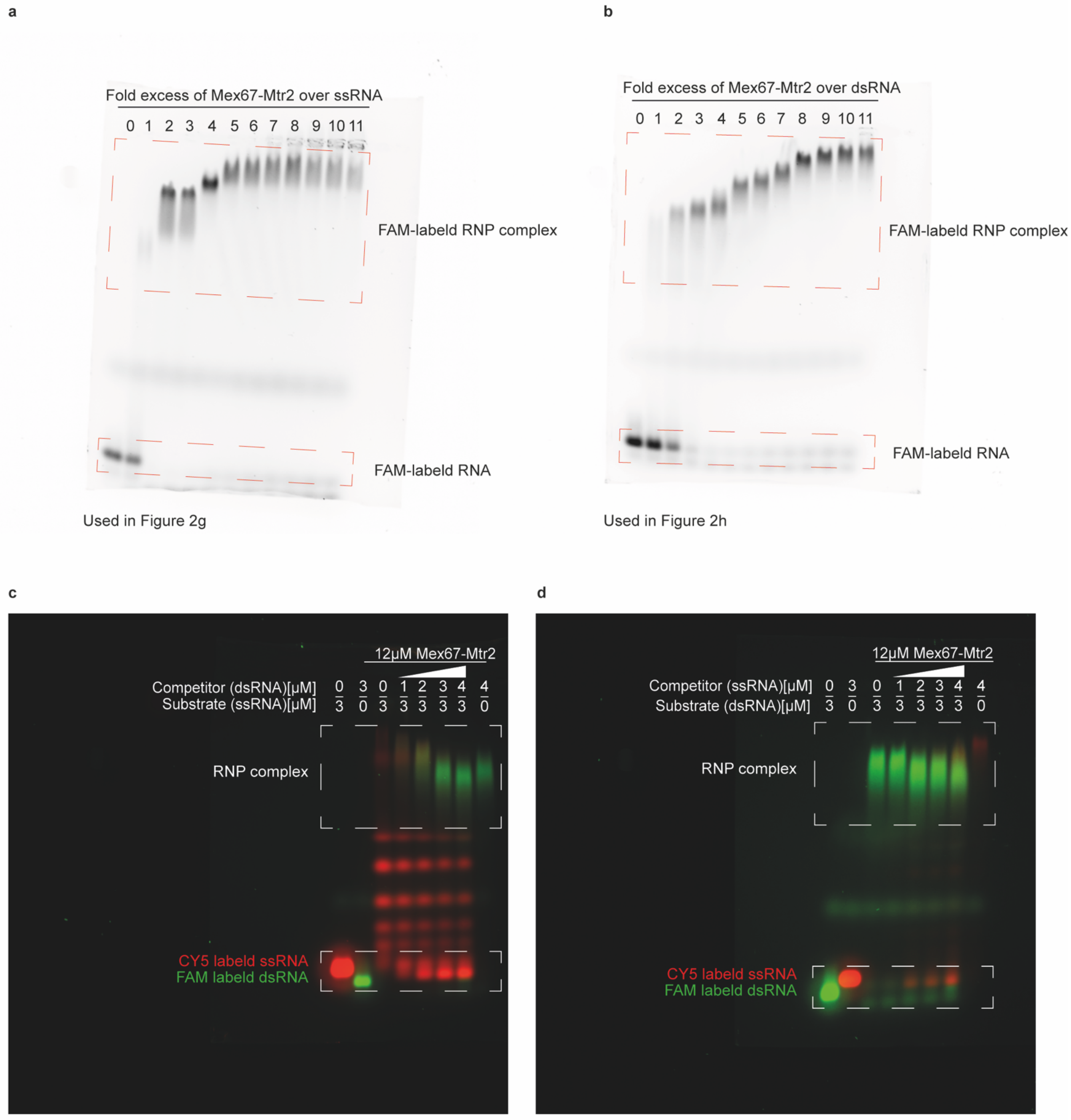

Used in Figure 2i

Used in Figure 2j

Coban et al., SI Figure 4

a

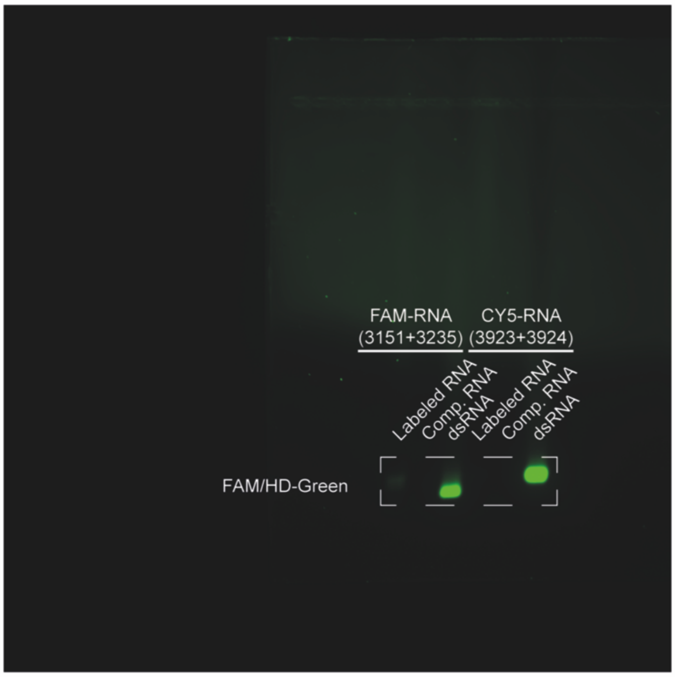

b

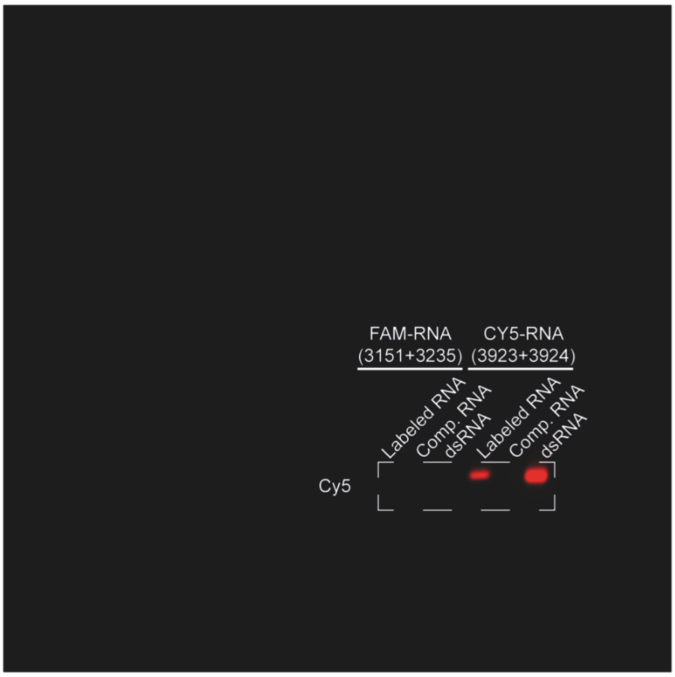

c

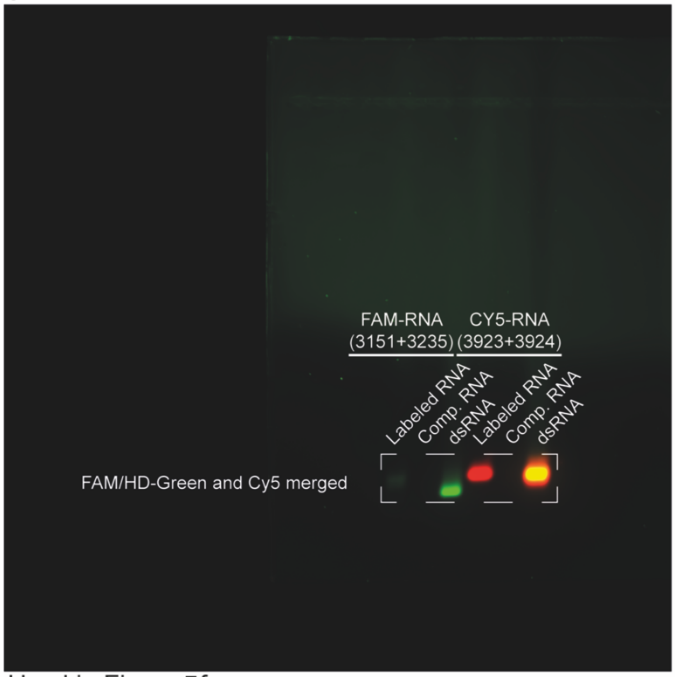

Used in Figure 7f

d

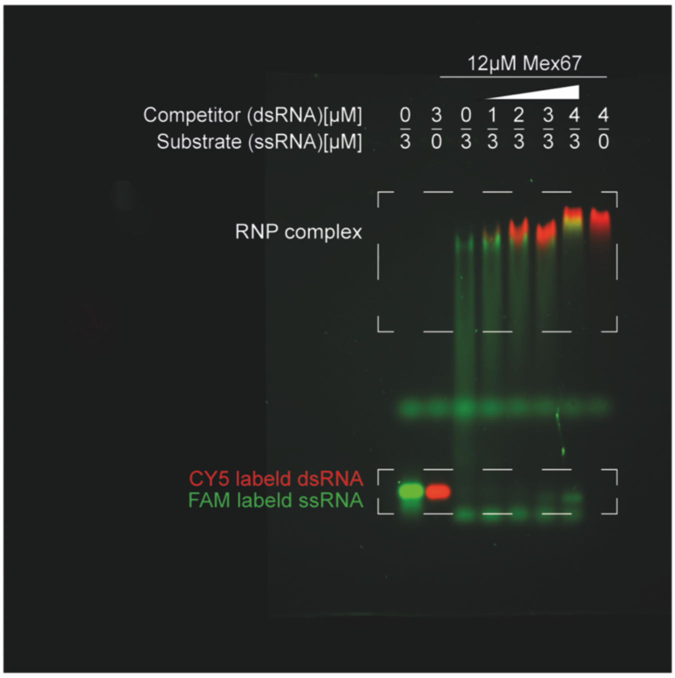

Used in Figure 7g

e

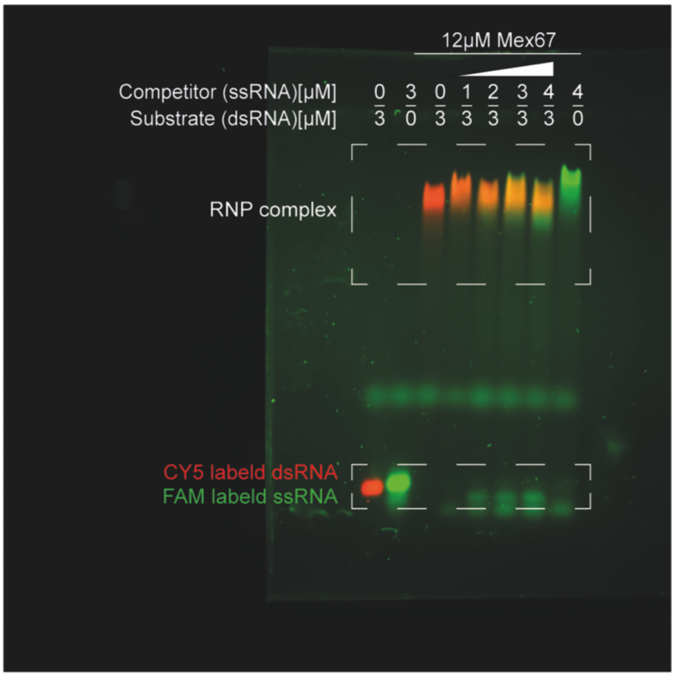

Used in Figure 7h

a

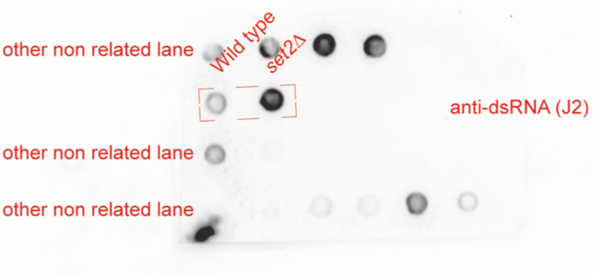

Used in Extended Figure 8c

b

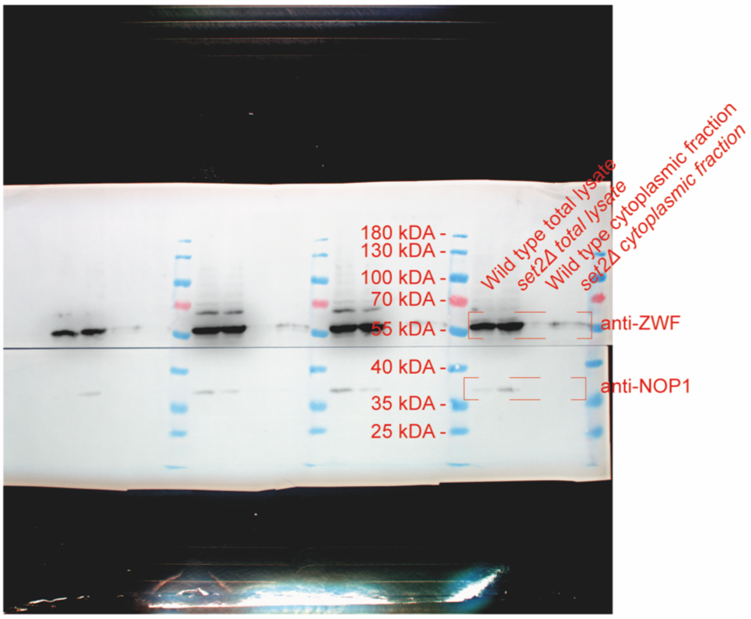

Used in Extended Figure 8f

c

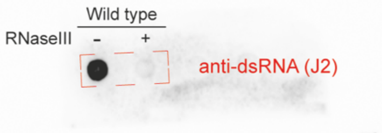

Used in Extended Figure 8h

d

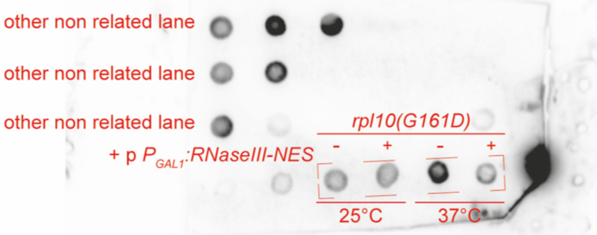

Used in Extended Figure 8n

a

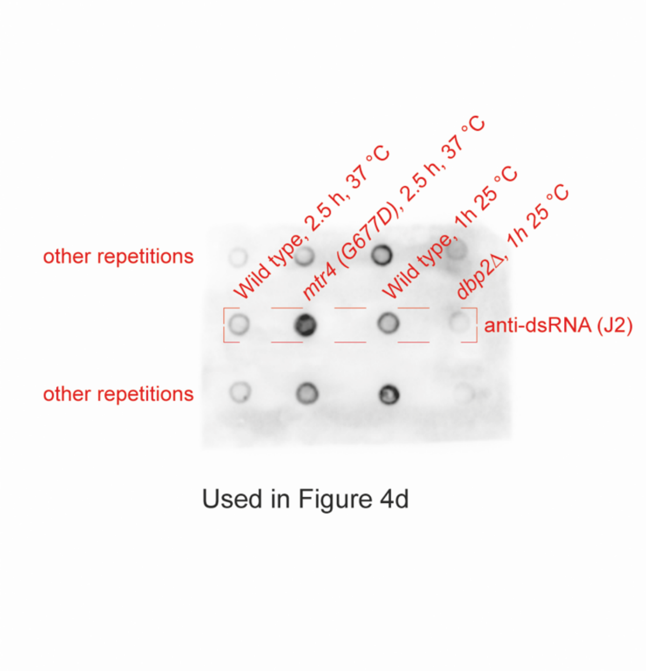

Used in Figure 4d

b

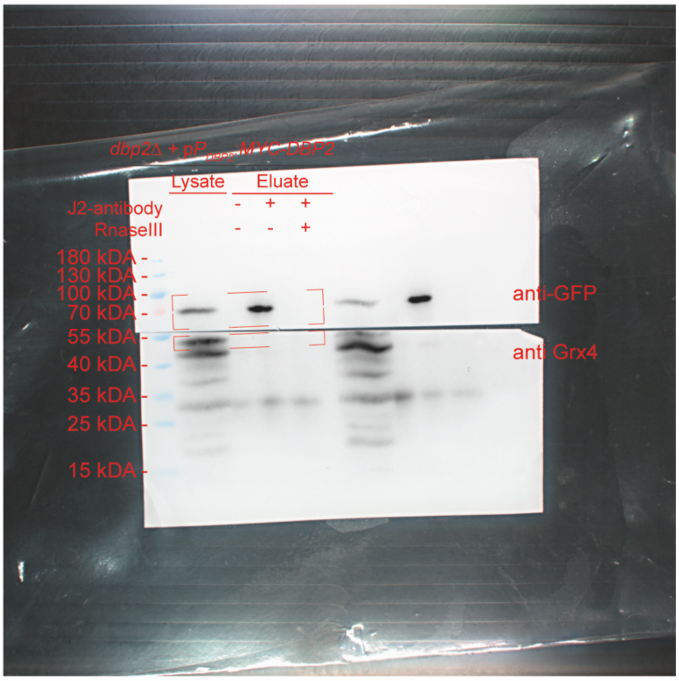

Used in Figure 4e

c

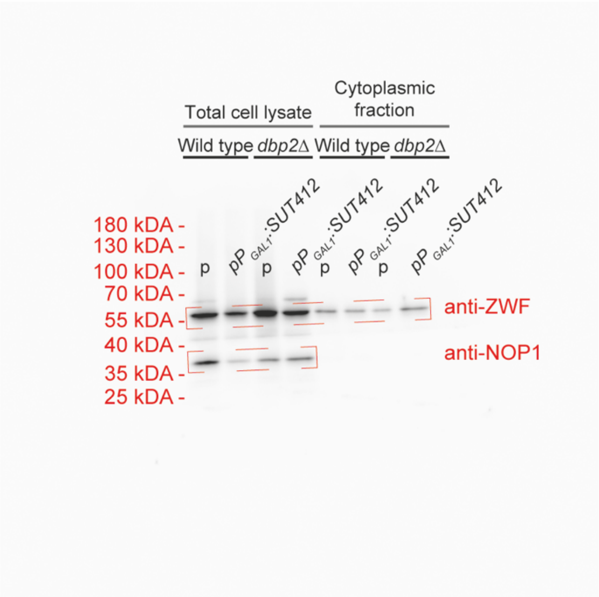

Extended Figure 8f

d

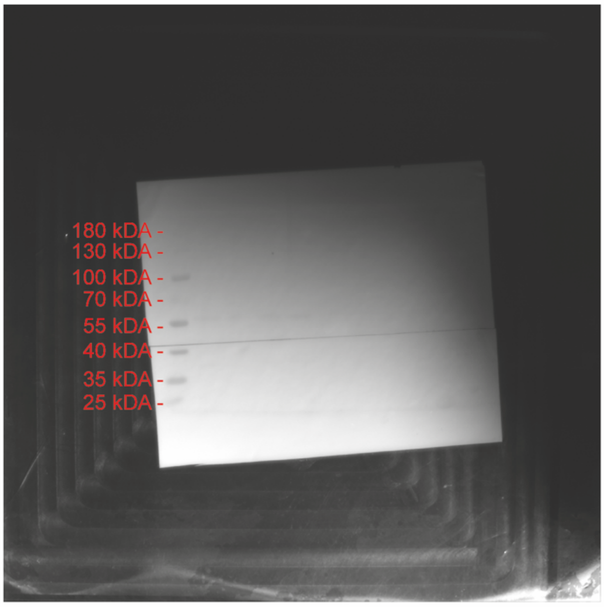

SI Table 1: Yeast strain

Number	Genotype	Source
HKY314	*MATa his3Δ1 leu2Δ0 met15Δ0 ura3Δ0*	Euroscarf
HKY863	*MATα rpl10::kanMX4 his3Δ1 leu2Δ0 ura3Δ0 lys2Δ0 prpl10(G161D)-GFP*	Baierlein C et al. (2013) Mol Cell Biol
HKY894	*MATα nmd3::kanMX4 his3 leu2 lys2 ura3 pnmd3-2*	Baßler et al. (2001) Mol. Cell
HKY1353	*MATa mex67::HIS3 xpo1::TRP1 ura3Δ0 pmex67-5 pxpo1-1*	Brune C et al. (2005) RNA
HKY1399	*MATα mtr4(G677D) ura3-52 leu2Δ1 his3Δ200*	This study
HKY1414	*MATa pho85::kanMX4 ura3Δ0 leu2Δ0 his3Δ1 met15Δ0*	Euroscarf
HKY1892	*MATα PHO85-GFP:HIS3MX6 his3Δ1 leu2Δ0 met15Δ0 ura3Δ0*	Euroscarf
HKY1898	*MATα set2::kanMX ura3Δ0 leu2Δ0 his3Δ1 met15Δ0*	Euroscarf
HKY2012	*MATα leu2-3, trp1-1, can1-100, ura3-1::EGFP::kanMX6 ade2-1 his3-11*	Drinnenberg et al. (2009) Science
HKY2013	*MATα leu2-3 trp1-1 can1-100 ura3-1::EGFP::kanMX6 ade2-1 his3-11 p*P_{TEF}*:DCR1(Scas) p*P_{TEF}*:AGO1(Scas)*	“
HKY2065	*DBP2/DBP2::kanMX4 MATa/MATα ura3Δ0/ura3Δ0 leu2Δ0/leu2Δ0 his3Δ1/his3Δ1 met15Δ0/MET15 LYS2/lys2Δ0*	Euroscarf
HKY2067	*MATα dbp2::kanMX4 ura3Δ0 leu2Δ0 his3Δ1 lys2Δ0*	This study

SI Table 2: Plasmids

Number	Genotype	Source
pHK697	*CEN URA RPS2-GFP*	Milkereit et al. (2003) JBC
pHK1716	*CEN URA* P_{GAL1}*:asPHO85*	This study
pHK1805	*CEN URA* P_{DBP2}*:MYC-DBP2*	This study
pHK1812	*CEN LEU* P_{GAL1}*:RNaseIII-NES-GFP*	This study
pHK1813	*CEN LEU* P_{GAL1}*:RNaseIII-NLS-GFP*	This study
pHK1814	*CEN LEU* P_{GAL1}*:RNaseIII-NES-NLS-GFP*	This study
pHK1815	*CEN LEU* P_{ADH1}*:RNaseIII-NES-GFP-GFP*	This study
pHK1798	*CEN URA* P_{GAL1}*:asPHO85-MYC(x15)*	This study
pHK1961	*CEN LEU* P_{GAL1}*:PHO85-GFP*	This study

SI Table 3: qPCR primer

Number	Sequence	Name
HK842	5'-CCAAGAACGTTTCTTGTTACAGACC-3'	*RPS6A* forward
HK843	5'-CGTCATCTTCCTTGGACAAACC-3'	*RPS6A* reverse
HK1002	5'-TGCTAAGGCTGTCGGTAAGG-3'	*TDH1* forward
HK1003	5'-TCAGAGGAGACAACGGCATC-3'	*TDH1* reverse
HK1024	5'-CAAGATTGCTGGTTACACCACC-3'	*RPS17A* forward
HK1025	5'-GTCTGTCTCTTTGGGCAGAAACG-3'	*RPS17A* reverse
HK2868	5‘-AGCACTAGTTGCGGTGAC-3‘	*HPF1* reverse
HK2870	5‘-TGAAGCCACAACCACTACTG-3‘	*HPF1* forward
HK2871	5‘-AGTTGTGGTGGTAGCTTCAG-3‘	*CSE1* reverse
HK2909	5‘-GCTGTATATGTCAGATGCGACTG-3‘	*FRE5* reverse
HK2911	5‘-CCTTCACTGCACACCACTAC-3‘	*FRE5* forward
HK2919	5‘-ACGTTAGTACATCAACCGGTG-3‘	*CSE1* forward
HK3001	5‘-ATTGTCGGTTGGACTAGCTG-3‘	*PRY3* reverse
HK3002	5‘-ACGCCATTACATCCGAGC-3‘	*PRY3* forward
HK3056	5'-TGAGTATCAAGCCACTGAGGTC-3'	*HEM15* forward
HK3057	5'-ACTGCCTTCTTCACGCCATC-3'	*HEM15* reverse
HK3919	5‘-AGCGTTCAACTAGCAGACCA-3‘	*GFP* forward
HK3920	5‘-GCCATGTGTAATCCCAGCAG-3‘	*GFP* reverse
HK3989	5‘-TCAAGTATCATTGGAAAGTAAAGAAC-3‘	*PHO85 intron* forward
HK3990	5‘-TTCTTTCATTAGGGAGATCTCAC-3‘	*PHO85 intron* reverse
HK3991	5‘-ACATTTTCAAGCGAAGTCG-3‘	*PHO85* forward
HK3992	5‘-CTGGATATTTGGGTTGTATTTG-3‘	*PHO85* reverse
HK4178	5‘-TGGAGGATGAGACTGGTAGTG-3‘	*SEG2* forward
HK4179	5‘-CCGGATCTCTCATTATCACG-3‘	*SEG2* reverse

SI Table 4: RNA-oligos used in EMSA

Number	Sequence	Name
HK3151	5‘-6FAM-UUUUUUUUAUUGCCUGGUUGCCUGGUUAUUUCUAUU-3‘	FAM-labeled RNA
HK3285	5‘-AAUAGAAAUAACCAGGCAACCAGGCAAUAAAAAAAA-3‘	RNA_complement_HK3151
HK3923	5‘-Cy5-UUAUAUGUCUUGUUCUCUUGUAUCUGUUCUUGUUGU-3‘	CY5-labeled RNA
HK3924	5‘-ACAACAAGAACAGAUACAAGAGAACAAGACAUAUAA-3‘	RNA_complement_HK3923

SI Table 5: probes used in FISH

Number	Sequence	Name
HK842	CY3-5'-CCATTAACATCACCATCTAATTCAACAAGAATTGGGACAACTCCAGTGAA-3'-CY3	*GFP1*
HK843	CY3-5'-CTTGACTTCAGCACGTGTCTTGTAGTTCCCGTCATCTTTGAAAAATATAG-3'-CY3	*GFP2*
HK1002	CY3-5'-TTCTTTAAAATCAATACCTTTTAACTCGATTCTATTAACAAGGGTATCAC-3'-CY3	*GFP3*
HK1003	CY3-5'-TCCGGGTATCTTGAAAAGCACTGAACACCATAAGTGAAAGTAGTGACAAG-3'-CY3	*GFP4*
HK1024	CY3-5'-CTCTTTTCGTTGGGATCTTTCGAAAGGGCAGATTGTGTGGACAGGTAATG-3'-CY3	*GFP5*
HK1025	CY3-5'-CTCTTTTCGTTGGGATCTTTCGAAAGGGCAGATTGTGTGGACAGGTAATG-3'-CY3	*GFP6*
HK2868	CY3-5'-CCTGTACATAACCTTCGGGCATGGCACTCTTGAAAAAGTCATGCCGTTTC-3'-CY3	*GFP7*
HK2870	CY3-5'-CCTTCACCCTCTCCACTGACAGAAAATTTG-3'-CY3	*GFP8*
HK2871	CY3-5'-GGCCATGGAACAGGTAGTTTTCCAGTAGTG-3'-CY3	*GFP9*
HK2909	CY3-5'-CCATGATGTATACATTGTGTGAGTTATAGT-3'-CY3	*GFP10*
HK2911	CY3-5'-CCATCTTCAATGTTGTGTCTAATTTTGAAG-3'-CY3	*GFP11*
HK2919	CY3-5'-CCATGTGTAATCCCAGCAGCTGTTACAAAC-3'-CY3	*GFP12*
HK3001	Alexa647-5'-CAAATCTTCTTCAGAAATCAACTTTTGTTC-3'-Alexa647	*MYC1*
HK3002	Alexa647-5'-CAAGTCTTCCTCGGAGATTAGCTTTTGTTC-3'-Alexa647	*MYC2*
HK3056	Alexa647-5'-CAAGTCTTCTTCTGAGATTAATTTTTGTTC-3'-Alexa647	*MYC3*
HK3057	CY3-5‘-TTTTTTTTTTTTTTTTTT-3‘-CY3	*oligo d(T)*

References to the tables

1 Baierlein, C. *et al.* Monosome formation during translation initiation requires the serine/arginine-rich protein Npl3. *Mol Cell Biol* **33**, 4811-4823 (2013). https://doi.org:10.1128/MCB.00873-13

2 Bassler, J. *et al.* Identification of a 60S preribosomal particle that is closely linked to nuclear export. *Mol Cell* **8**, 517-529 (2001).

3 Brune, C., Munchel, S. E., Fischer, N., Podtelejnikov, A. V. & Weis, K. Yeast poly (A) -binding protein Pab1 shuttles between the nucleus and the cytoplasm and functions in mRNA export Yeast poly (A) -binding protein Pab1 shuttles between the nucleus and the cytoplasm and functions in mRNA export. *Rna-a Publication of the Rna Society*, 517-531 (2005). https://doi.org:10.1261/rna.7291205.family

4 Drinnenberg, I. A. *et al.* RNAi in budding yeast. *Science* **326**, 544-550 (2009). https://doi.org:10.1126/science.1176945

5 Milkereit, P. *et al.* A Noc complex specifically involved in the formation and nuclear export of ribosomal 40 S subunits. *J Biol Chem* **278**, 4072-4081 (2003). https://doi.org:10.1074/jbc.M208898200

6 Hackmann, A. *et al.* Quality control of spliced mRNAs requires the shuttling SR proteins Gbp2 and Hrb1. *Nat Commun* **5**, 3123 (2014). https://doi.org:10.1038/ncomms4123
