# Supplementary material for: dsRNA formation leads to preferential nuclear export and gene expression
Source: Nature. 2024 Jun 19;631(8020):432–8. doi: 10.1038/s41586-024-07576-w (PMC11236707; doi:10.1038/s41586-024-07576-w)
Supplement: Supplementary file 2 — Reporting Summary [file 41586_2024_7576_MOESM2_ESM.pdf]

nature portfolio

Corresponding author(s): Prof. Dr. Heike Krebber

Last updated by author(s): 2024.05.10

# Reporting Summary

Nature Portfolio wishes to improve the reproducibility of the work that we publish. This form provides structure for consistency and transparency in reporting. For further information on Nature Portfolio policies, see our Editorial Policies and the Editorial Policy Checklist.

## Statistics

For all statistical analyses, confirm that the following items are present in the figure legend, table legend, main text, or Methods section.

| n/a | Confirmed | |
|---|---|---|
| ☐ | ☒ | The exact sample size (*n*) for each experimental group/condition, given as a discrete number and unit of measurement |
| ☐ | ☒ | A statement on whether measurements were taken from distinct samples or whether the same sample was measured repeatedly |
| ☐ | ☒ | The statistical test(s) used AND whether they are one- or two-sided<br>*Only common tests should be described solely by name; describe more complex techniques in the Methods section.* |
| ☒ | ☐ | A description of all covariates tested |
| ☒ | ☐ | A description of any assumptions or corrections, such as tests of normality and adjustment for multiple comparisons |
| ☐ | ☒ | A full description of the statistical parameters including central tendency (e.g. means) or other basic estimates (e.g. regression coefficient) AND variation (e.g. standard deviation) or associated estimates of uncertainty (e.g. confidence intervals) |
| ☒ | ☐ | For null hypothesis testing, the test statistic (e.g. *F*, *t*, *r*) with confidence intervals, effect sizes, degrees of freedom and *P* value noted<br>*Give P values as exact values whenever suitable.* |
| ☒ | ☐ | For Bayesian analysis, information on the choice of priors and Markov chain Monte Carlo settings |
| ☒ | ☐ | For hierarchical and complex designs, identification of the appropriate level for tests and full reporting of outcomes |
| ☐ | ☒ | Estimates of effect sizes (e.g. Cohen's *d*, Pearson's *r*), indicating how they were calculated |

*Our web collection on statistics for biologists contains articles on many of the points above.*

## Software and code

Policy information about availability of computer code

| | |
|---|---|
| Data collection | Single read (50 bp) sequencing was conducted using a HiSeq 4000 (Illumina). Fluorescence images were transformed to BCL files with the Illumina BaseCaller software (version 3.6.3) and samples were demultiplexed to FASTQ files with bcl2fastq (version 2.17). |
| Data analysis | Sequences were aligned to the genome reference sequence of Saccharomyces cerevisiae (sacCer3, obtained from UCSC, https://hgdownload.cse.ucsc.edu/goldenPath/sacCer3/bigZips/) using the STAR software (49; version 2.5) allowing for 2 mismatches. Subsequently, abundance measurement of reads overlapping with exons or introns was conducted with featureCounts (50, subread version 1.5.0-p1, Ensembl (EF4.68) supplemented with the coordinates of UTRs, CUTs and SUTs 22,51,52 and Xrn1-sensitive unstable transcripts 3,29 Data was processed in the R/Bioconductor environment (www.bioconductor.org, R version 3.6.1) using the DESeq2 package (53; version 1.24.0). The sequencing data and abundance measurement files have been submitted to the NCBI Gene Expression Omnibus (GEO) database. For null hypothesis testing the Wald test was used with multiple comparisons adjustments using the Benjamini and Hochberg method. In downstream analysis only transcripts with an average count above 40 were considered.<br>Overlapping features respectively sense and antisense pairs were identified with BEDTools intersect (version 2.3.1) 54 requiring overlaps to occur on the opposite strand with a minimum overlap of 0.5.<br>For gene coverage of RNAi degradation products, the reads were trimmed using Cutadapt (version 2.1) 55 and aligned to the reference genome with TopHat2 (version 2.1.1) 56. For gene coverage the geneBody_coverage module of the RSeQC package was used (version 2.6.4) 57. The input BED file was filtered by lncRNA classes (SUT, CUT or XUT) or by RNA enrichment in RNAi-seq. Overlapping features on the same strand were excluded. |

For manuscripts utilizing custom algorithms or software that are central to the research but not yet described in published literature, software must be made available to editors and reviewers. We strongly encourage code deposition in a community repository (e.g. GitHub). See the Nature Portfolio guidelines for submitting code & software for further information.

# Data

Policy information about availability of data

All manuscripts must include a data availability statement. This statement should provide the following information, where applicable:

- Accession codes, unique identifiers, or web links for publicly available datasets
- A description of any restrictions on data availability
- For clinical datasets or third party data, please ensure that the statement adheres to our policy

Fractionation-RNA-Seq data have been deposited at the NCBI gene expression omnibus (GEO; www.ncbi.nlm.nih.gov/geo/) with the GEO accession number GSE188455. J2-RIP-seq data can be accessed with accession number GSE252951. RNA-seq of Cells exposed to 0.6M NaCl was provided 34 under the accession number GSE89554. RNAi-seq data has been deposited by Wery and colleagues under the accession number GSE64090. Dbp2 iCLIP and Structure-Seq data can be found under the accession number GSE106479 provided 45.

# Field-specific reporting

Please select the one below that is the best fit for your research. If you are not sure, read the appropriate sections before making your selection.

☒ Life sciences ☐ Behavioural & social sciences ☐ Ecological, evolutionary & environmental sciences

For a reference copy of the document with all sections, see nature.com/documents/nr-reporting-summary-flat.pdf

# Life sciences study design

All studies must disclose on these points even when the disclosure is negative.

| | |
|---|---|
| Sample size | All statistically analysed experiments were independently repeated at least three times and determined according to standard molecular biology procedures. |
| Data exclusions | Data were excluded from analysis only due to technical failure. Western blot analysis of co-immunoprecipitation, RNA co-immunoprecipitation and cytoplasmic fractionation demonstrated the integrity of these experiments. |
| Replication | All experiments were reproducible and confirmed by statistical analysis as described. At least three independent replicates were performed for statistically analysed experiments. Samples for RNA sequencing were tested prior to sequencing. For cytoplasmic fractionation, Western blots were prepared and the distribution of known RNAs such as rRNAs and snoRNAs was tested by qPCR. For J2-RIP-seq, known dsRNAs were tested by qPCR. Validated samples were then sent for RNA sequencing. |
| Randomization | No randomization was used. Samples were prepared from a minimum of 1x10^8 cells and appropriate controls were included. |
| Blinding | Blinding was not performed. Quantification of fluorescent signal was performed on all intact cells of an image section from three independent replicates. Acquisition and quantification were performed by multiple researchers. |

# Reporting for specific materials, systems and methods

We require information from authors about some types of materials, experimental systems and methods used in many studies. Here, indicate whether each material, system or method listed is relevant to your study. If you are not sure if a list item applies to your research, read the appropriate section before selecting a response.

## Materials & experimental systems

| n/a | Involved in the study | |
|---|---|---|
| ☐ | ☒ | Antibodies |
| ☐ | ☒ | Eukaryotic cell lines |
| ☒ | ☐ | Palaeontology and archaeology |
| ☒ | ☐ | Animals and other organisms |
| ☒ | ☐ | Human research participants |
| ☒ | ☐ | Clinical data |
| ☒ | ☐ | Dual use research of concern |

## Methods

| n/a | Involved in the study | |
|---|---|---|
| ☒ | ☐ | ChIP-seq |
| ☒ | ☐ | Flow cytometry |
| ☒ | ☐ | MRI-based neuroimaging |

## Antibodies

| | |
|---|---|
| Antibodies used | anti-Nop1, Sanata Cruz, sc-57940, 28F2; anti-Myc, Santa Cruz, sc-789, A-14; anti-Yra1, Santa Cruz, yc-14; anti-Zwf1, Prof. Dr. Ulrich Mühlenhoff; anti-Hem15, Prof. Dr. Ulrich Mühlenhoff; anti-Aco1, Prof. Dr. Ulrich Mühlenhoffl; anti-Grx4, Prof. Dr. Ulrich Mühlenhoffl; anti-dsRNA, Jena Bioscience (Scicons), RNT-SCI-10010200, J2; anti-GFP, Chromotek, PABG1-100, PABG1; goat anti-mouse HRP |

| | |
|---|---|
| | conjugated, Dianova, 115-035-146; goat anti-rabbit HRP conjugated, Dianova, 111-035-144; goat anti-mouse Cy3 conjugated, Dianova, 115-165-146 |
| Validation | anti-dsRNA: Purity/Identity: Reducing and Non-reducing SDS-PAGE, Activity: AN-ELISA (relative activity compared to reference J2), Schönborn et al. (1991) Monoclonal antibodies to double-stranded RNA as probes of RNA structure in crude nucleic acid extracts. Nucleic Acids Res.19: 2993., Extended Data Fig. 2a, RRID:AB_2651015<br>anti-GFP: Purity: Affinity-purified antibody, Tested applications: Western Blot and Immunofluorescence, RRID:AB_2749857<br>anti-Nop1: Tested in Western blot, Source. Nop1p (28F2) is a mouse monoclonal antibody raised against a nuclear preparation of S. cerevisiae origin. (Santa Cruz)<br>anti-Yra1: Tested in Western blot (Santa Cruz)<br>anti-Myc: Tested apllication: Western blot; RRID:AB_631275<br>anti-Zwf1, anti-Hem15, anti-Aco1, anti-Grx4: Tested apllication: Western blot of recombinant proteins |

## Eukaryotic cell lines

Policy information about cell lines

| | |
|---|---|
| Cell line source(s) | HKY314: BY4741 Wild type, Euroscarf<br>HKY894: nmd3-2, Brune et al. 2005<br>HKY863; rpl10(G161D), Baierlain et al., 2013<br>HKY1353: mex67-5 xpo1-1, Gadal et al., 2001<br>HKY1399: mtr(G667D), Hackmann et al., 2014<br>HKY1414: pho85::kanMX4; Eurpscarf<br>HKY1892: PHO85-GFP; Euroscarf<br>HKY1898: set::kanMX4, Euroscarf<br>HKY2012: W303 Wild type, Drinnenberg et al., 2009<br>HKY2013: W303 pDCR1 pAGO1, Drinnenberg et al., 2009<br>HKY2065: DBP2/dbp2::kanMX4, Euroscarf<br>HKY2067: dbp2::kanMX4, this study |
| Authentication | Each strain was verified via growth on selective plates and by seqncing of specific genes and markers<br>HKY863, HKY1353 and HKY2067 were authenticated via growthtest analysis and polyA-FISH |
| Mycoplasma contamination | *Confirm that all cell lines tested negative for mycoplasma contamination OR describe the results of the testing for mycoplasma contamination OR declare that the cell lines were not tested for mycoplasma contamination.* |
| Commonly misidentified lines<br>(See ICLAC register) | *Name any commonly misidentified cell lines used in the study and provide a rationale for their use.* |
